# Focus on gut microbes: new direction in cancer treatment

**DOI:** 10.3389/fonc.2025.1505656

**Published:** 2025-08-29

**Authors:** Lingshan Liao, Mengying Zeng, Debei Liu, Yuxing He, Wei Du, Yanlin Cao

**Affiliations:** ^1^ Department of Pathology, Changde Hospital, Xiangya School of Medicine, Central South University, Changde, China; ^2^ Department of Clinical Laboratory, Changde Hospital, Xiangya School of Medicine, Central South University, Changde, China

**Keywords:** gut microbes, tumor therapy, tumor microenvironment, drug resistance, immunotherapy

## Abstract

Gut microbes are emerging as critical regulators in cancer therapy, influencing the efficacy and toxicity of radiotherapy, chemotherapy, immunotherapy, targeted therapy, Traditional Chinese Medicine, and rehabilitation interventions. Acting through metabolic reprogramming, immune modulation, DNA damage, and tumor microenvironment remodeling, specific microbial taxa and their metabolites can either enhance or hinder treatment outcomes. However, these interactions are highly context-dependent and shaped by individual factors such as diet, geography, and host immunity. While microbial interventions such as probiotics, fecal microbiota transplantation, and engineered bacteria show promise, their translation into precise and safe clinical applications remains limited by interindividual variability, regulatory hurdles, and incomplete mechanistic understanding. Future efforts should focus on defining high-evidence microbial signatures, clarifying causal mechanisms, and developing personalized microbiome-based therapeutic strategies, potentially integrated with nanotechnology. This review underscores the need for interdisciplinary approaches to harness gut microbiota as co-targets in cancer treatment.

## 1 Introduction

The gut, as the body’s largest immune organ, plays a central role in immune surveillance and tolerance. Constantly exposed to dietary and microbial antigens, it also serves as a potential entry point for pathogens. Approximately 30–40 dominant bacterial species shape the adult gut microbiota, whose composition is dynamic and influenced by diet, smoking, medications (e.g., antibiotics), probiotics, and host physiology (1, 2). Along the gastrointestinal tract, bacterial density increases distally, with anaerobes dominating the colon. Microbial colonization begins at birth and stabilizes by age two, facilitating mucosal immune maturation and the balance between inflammation and immune tolerance (3, 4).

Gut microbiota contributes to host health by regulating nutrient metabolism, xenobiotic detoxification, epithelial development, immune modulation, and resistance to pathogen colonization (5–10). Conversely, dysbiosis can promote malignancies, notably colorectal and hepatocellular carcinomas (11). In rodent models, germ-free or antibiotic-treated conditions have revealed microbiota-driven tumorigenesis, independent of inflammation (12). Microbial biofilms may also reshape the tumor microenvironment (TME) by promoting metabolic cross-talk and immune evasion (13–15).

Beyond tumorigenesis, gut microbes profoundly influence the efficacy and toxicity of anticancer therapies, especially immunotherapy (15, 16). The TME has emerged as a critical determinant of therapy response and is shaped by microbial-derived metabolites and immune signaling (17–20). Specific microbes enhance immune cell infiltration and antigen presentation, while others hinder treatment through immune suppression. Novel strategies such as probiotic supplementation, fecal microbiota transplantation (FMT), bacterial engineering, and phage therapy are under investigation for enhancing therapeutic outcomes (21–24).

Despite rapid progress, challenges remain. Interindividual variability in microbiota composition, limited mechanistic understanding, and regulatory constraints hinder clinical translation. Moreover, the interplay between gut microbes, host immunity, and cancer remains complex and context-dependent. This review provides an updated synthesis of how gut microbiota modulate responses to multiple cancer therapies, including radiotherapy, chemotherapy, targeted therapy, immunotherapy, Traditional chinese medicine (TCM), and rehabilitation interventions. We highlight key mechanisms—ranging from metabolic reprogramming and immune modulation to TME remodeling—and discuss their translational potential. Looking forward, personalized microbiota-based interventions and interdisciplinary innovations such as AI-driven microbial profiling may pave the way for safer and more effective cancer treatment strategies.

## 2 Influence of gut microbes on multiple antitumor therapies

### 2.1 Radiation therapy and chemotherapy

Radiotherapy and chemotherapy remain cornerstone treatments for various malignancies. However, increasing evidence highlights the gut microbiota as a critical modulator of both their therapeutic efficacy and associated toxicities.

Radiation-induced damage not only alters tumor tissues but also disrupts intestinal microbial homeostasis. Certain microbial populations can exacerbate the toxicity of radiation therapy. Animal studies have demonstrated that radiation-induced alterations in the gut microbiota promote the secretion of interleukin-1β (IL-1β) and the generation of reactive oxygen species (ROS), which in turn disrupt intestinal tight junctions and amplify inflammatory processes, thereby further aggravating mucosal inflammation in mice (25, 26). These effects compromise quality of life and may necessitate dose reductions or treatment suspension. Conversely, specific commensal strains—particularly those with anti-inflammatory or mucosal barrier-protective properties—can mitigate such adverse effects (27). Certain intestinal microorganisms—such as *Lactobacillus rhamnosus, Lactobacillus acidophilus, Bifidobacterium*, members of the families *Lachnospiraceae and Enterococcaceae, as well as Akkermansia*—have been reported to mitigate the side effects of radiation therapy (27, 28).

Clinical studies reinforce these findings. In a large double-blind, placebo-controlled trial, Delia et al. demonstrated that probiotic supplementation significantly reduced radiation-induced diarrhea in postoperative cancer patients (29). Similarly, Sharma et al.found that probiotics lowered the incidence of grade III-IV oral mucositis in patients receiving radiotherapy for head and neck cancers, improving treatment completion rates (30). These results support the integration of targeted probiotic interventions into radiotherapy regimens to reduce complications and improve therapeutic adherence.

Gut microbes can influence chemotherapy through multiple mechanisms, including drug metabolism, immune modulation, and barrier integrity maintenance. Numerous studies have demonstrated that gut microbiota can modulate the efficacy of chemotherapeutic agents (31). Specific bacterial species, such as *Bacteroides fragilis* and *Lactobacillus acidophilus*, can influence the bioavailability and antitumor activity of chemotherapeutic drugs through metabolic interactions. For example, Bacteroides fragilis has been shown to metabolize agents like 5-fluorouracil (5-FU), thereby affecting its therapeutic impact (32). Additionally, oxaliplatin (OXP) chemotherapy has been reported to enhance local immune responses by modulating the ileal microbiota, ultimately improving its clinical antitumor efficacy. In contrast, cisplatin can induce alterations in commensal gut bacteria, exacerbating mucosal injury, increasing tumor burden, and triggering systemic inflammation. Notably, these adverse effects can be reversed by the administration of *Lactobacillus acidophilus (* 33).

Microbial metabolites also play crucial roles. Butyrate enhances gemcitabine-induced apoptosis, while microbial β-glucuronidase can reactivate irinotecan’s active metabolite SN-38, leading to toxicity—an effect reversible by co-administering β-glucuronidase inhibitors. Conversely, microbial enzymes like cytidine deaminase may inactivate gemcitabine, promoting drug resistance in pancreatic and colorectal cancers.

**Figure 1:**
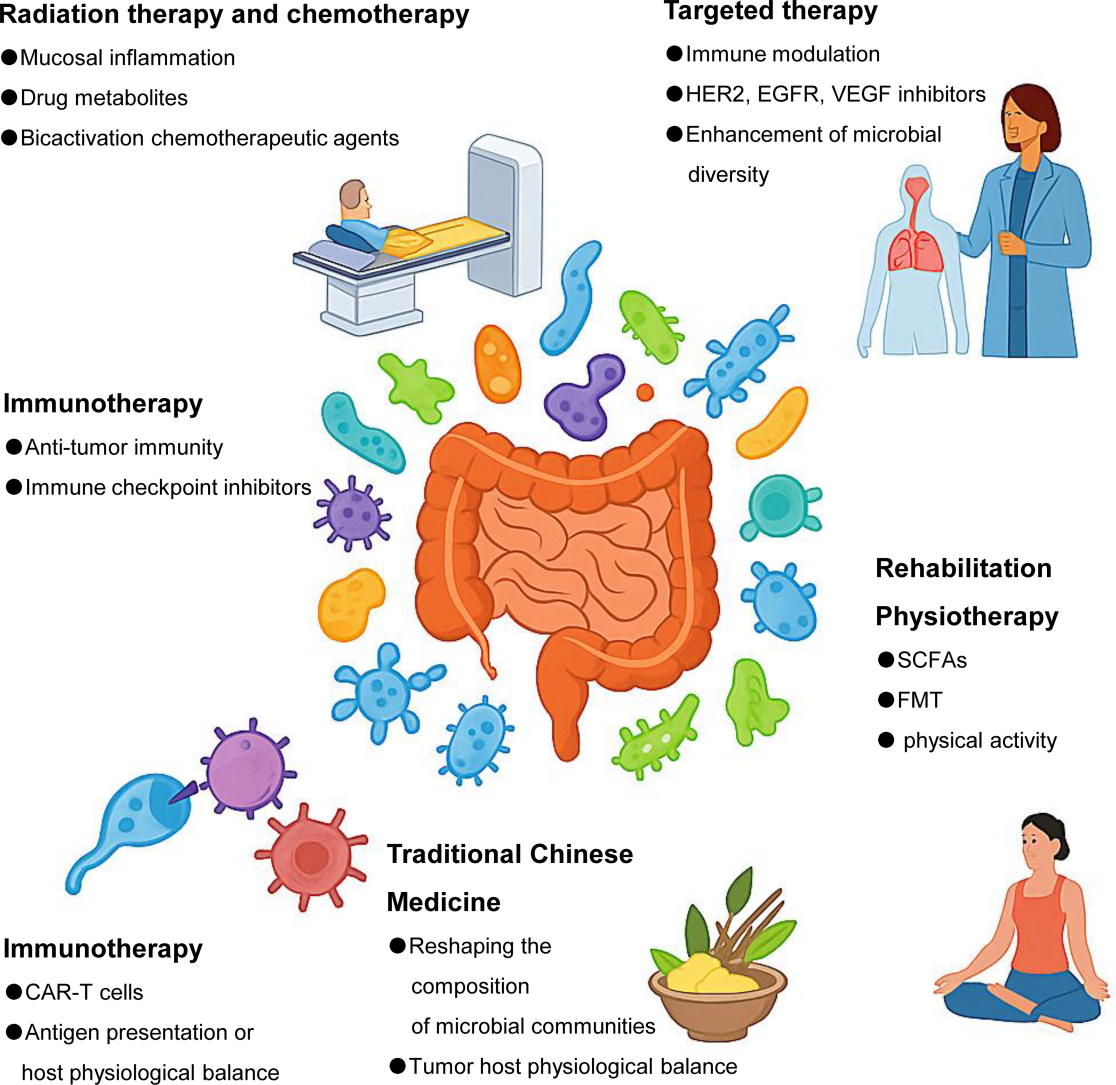
Gut microbes are associated with a wide range of tumor therapies. Gut microbes influence multiple aspects of cancer treatment, including radiotherapy, chemotherapy, immunotherapy, targeted therapy, TCM, and rehabilitation strategies such as dietary intervention and FMT. Specific microbial taxa can modulate treatment efficacy and toxicity through diverse mechanisms, such as regulating immune responses, altering drug metabolism, maintaining intestinal barrier integrity, or shaping the tumor microenvironment.

Gut microbiota act as both mediators and modulators of chemo-radiotherapeutic outcomes (**Figure 1**). Their dual role in enhancing efficacy and limiting toxicity opens promising avenues for microbiota-informed oncologic strategies. However, interpatient variability, context-dependent responses, and incomplete mechanistic understanding remain significant challenges. Future research should aim to identify predictive microbial signatures, explore metabolite-host-drug interactions in depth, and design microbiome-based adjuvant therapies tailored to individual tumor types and treatment protocols.

### 2.2 Targeted therapy

Molecular targeted therapies, designed to interfere with specific oncogenic signaling pathways, have significantly improved cancer treatment (34). However, recent studies reveal that gut microbiota can modulate the efficacy of several commonly used targeted agents, including trastuzumab (HER2 inhibitor), cetuximab (EGFR inhibitor), and bevacizumab (VEGF inhibitor) (35). A phase I trial (NCT03772899) evaluated healthy donor fecal microbiota transplantation (FMT) combined with PD-1 inhibitors in 20 treatment-naïve advanced melanoma patients. FMT alone was safe, with no grade 3 events; combined therapy led to a 65% response rate (including 20% complete responses) but 25% grade 3 immune-related adverse events. Microbiome analysis showed donor strain engraftment and increased similarity over time only in responders, alongside enrichment of immunogenic and reduction of harmful bacteria. Mouse models confirmed enhanced anti-PD-1 efficacy, supporting further investigation of FMT as an adjunct to immunotherapy (36).

In HER2-positive breast cancer, low intestinal abundances of Trichoderma, Zygomycetes, Bifidobacterium, and Prevotella were found in trastuzumab-nonresponsive patients. In murine models, antibiotic-induced microbiota depletion reduced trastuzumab efficacy by impairing CD4^+^ T cell and granzyme B^+^ cell infiltration, dendritic cell activation, and IL-12 secretion within tumors (37). These findings suggest that gut microbes may enhance trastuzumab’s therapeutic effect through immune modulation.

Similarly, gut microbiota diversity has been associated with improved outcomes in colorectal cancer (CRC) patients treated with cetuximab or bevacizumab. In contrast, high levels of Klebsiella pneumoniae, Lactobacillus, Bifidobacterium, and Clostridium perfringens correlated with disease progression and poorer prognosis, indicating that not all bacteria exert beneficial effects (38).

In prostate cancer, the microbiome’s influence extends to hormonal therapy. Patients receiving androgen axis-targeted therapies (e.g., bicalutamide, enzalutamide, abiraterone) exhibited enriched bacterial taxa capable of steroid biosynthesis (39). Notably, Coccidioides and Mycobacterium species were more abundant in castration-resistant patients and were shown to convert androgen precursors into active forms, thereby compromising the efficacy of androgen deprivation therapy (40).

These studies underscore the role of gut microbiota not only as passive biomarkers but also as active modulators of targeted therapy response. Their immunoregulatory capacity and metabolic flexibility—such as influencing cytokine signaling or steroid metabolism—can either enhance or undermine treatment efficacy. Future directions should include identifying microbial signatures predictive of response to targeted agents and exploring microbiome modulation as a strategy to overcome resistance in precision oncology (**Figure 1**).

### 2.3 Immunotherapy

Immunotherapy has revolutionized cancer treatment, offering durable responses across various malignancies through approaches such as immune checkpoint inhibitors (ICIs), CAR-T cells, oncolytic viruses, cancer vaccines, and cytokine therapies (41–43). However, increasing evidence reveals that gut microbiota critically shape both the efficacy and toxicity of these immune-based therapies (44, 45).

Clinical and preclinical studies have demonstrated that gut microbes modulate antitumor immunity, particularly in T-cell-mediated therapies (17, 33). Disruption of the gut microbiota, such as through antibiotics, has been shown to impair responses to CAR-T therapy. For instance, patients treated with antibiotics exhibited increased tumor burden and systemic inflammation, while those colonized with Bifidobacterium longum and peptidoglycan-producing microbes prior to CAR-T therapy showed improved 6-month survival and reduced tumor progression (46).

Pioneering studies by Sivan et al. (47) first identified the microbiota-dependency of ICI efficacy. Bifidobacterium intestinalis and Lactobacillus casei paracasei enhanced CD8^+^ T-cell infiltration and dendritic cell (DC) activation, boosting anti-PD-L1 activity in colorectal cancer models (38,39). Likewise, B. longum improved anti-PD-L1 responses in melanoma by promoting tumor-specific CD8^+^ T-cell effector functions (48).

Subsequent studies confirmed that Bifidobacterium pseudomallei and B. bifidum similarly enhanced ICI efficacy across multiple tumor models through oral administration or modulation of DC function (49). In addition, Clostridium perfringens activated the STING pathway, promoting PD-L1 expression and IFN-γ^+^ CD8^+^ TIL accumulation, further sensitizing tumors to PD-L1 blockade (50). Interestingly, the efficacy of ICIs may be influenced not only by microbial composition but also by tumor type, as different malignancies induce distinct microbiota alterations that can either enhance or impair immunotherapeutic response (51).

Gut microbiota plays a dual role in immunotherapy: they can augment antitumor immunity or contribute to resistance. Key taxa such as *Bifidobacterium* spp. *and Clostridium* spp. exert their effects through modulation of antigen presentation, cytokine signaling, and T-cell recruitment. Given the complex and context-dependent interactions, future work should focus on identifying microbial biomarkers predictive of ICI responsiveness and developing microbial adjuvants or preconditioning strategies to optimize immune-based therapies (**Figure 1**).

### 2.4 Traditional Chinese medicine

In recent years, cancer treatment strategies have become increasingly diversified, ranging from modern immunotherapies to TCM (52). While these approaches differ significantly in their theoretical foundations, mechanisms of action, and clinical application, they also exhibit potential complementarities. ICIs, a hallmark of modern immunotherapy, offer targeted interventions against tumor immune evasion with robust clinical efficacy and scientific reproducibility. However, they are often associated with variable patient responses and immune-related adverse events. In contrast, TCM adopts a holistic and multi-targeted approach, emphasizing the principles of “reinforcing the body’s vital energy and eliminating pathogenic factors” and “harmonizing organ function.” It has shown promise in enhancing immune function, mitigating treatment-related toxicity, and improving patients’ quality of life. Emerging evidence suggests that TCM may enhance immunotherapy outcomes by modulating the gut microbiota and reducing systemic inflammation, indicating its potential as a valuable adjunct to modern treatments. Future research exploring the synergistic mechanisms of microbiota regulation and immune modulation between TCM and immunotherapy may pave the way for integrated cancer treatment paradigms.

Traditional chinese medicine has demonstrated notable efficacy in complex diseases, including malignancies, where it contributes to symptom relief, immune regulation, and prevention of metastasis and recurrence (53). Recent studies highlight a bidirectional interaction between TCM and gut microbiota, positioning this interplay as a potential mediator of TCM’s therapeutic effects in oncology.

On one hand, TCM can reshape gut microbial composition and metabolism, thereby restoring host physiological balance and alleviating tumor-promoting conditions. Herbal compounds may promote beneficial taxa and suppress pathogenic ones, contributing to anti-inflammatory and antitumor effects. On the other hand, gut microbes are involved in the biotransformation of TCM components, enhancing the bioavailability and activity of pharmacologically relevant metabolites. However, some microbial species may antagonize TCM efficacy by degrading active compounds or interfering with their absorption.

Mechanistically, TCM exerts its antitumor effects through modulation of host–microbiota axes, including the gut–liver, gut–brain, and gut–immune pathways, thereby influencing endocrine and immune networks (54). This modulation helps to disrupt the tumor-favorable microenvironment and restore systemic immune homeostasis.

Specific herbal formulations further exemplify this mechanism. For instance, the Paeonia lactiflora Soft Liver Combination significantly reduced Mycobacterium avium abundance, while Jiawei Yuxuan decoction altered gut microbial profiles and regulated key metabolites—such as primary bile acids and IFN-γ—in a hepatocellular carcinoma model, thereby enhancing antitumor immunity (17). These findings support the microbiota-dependent therapeutic potential of TCM in liver and colorectal cancers.

The gut microbiota–TCM axis represents a promising frontier in integrative oncology. However, the dualistic nature of this interaction—where gut microbes can both enhance and hinder TCM efficacy—necessitates careful characterization of host-microbe-drug dynamics. Future research should aim to identify microbial biomarkers predictive of TCM responsiveness, optimize herbal compound formulation for microbiota compatibility, and avoid unintended microbial interference. Particularly in palliative care, where TCM offers symptomatic relief with minimal toxicity, microbiota-informed TCM strategies may become valuable adjuncts to mainstream cancer therapies (**Figure 1**).

### 2.5 Rehabilitation physiotherapy

Rehabilitation therapies for cancer patients increasingly recognize the role of gut microbiota as a modifiable factor influencing prognosis, treatment tolerance, and quality of life. Modulating the gut microbial ecosystem—through dietary interventions, probiotics, FMT, physical activity, or circadian rhythm regulation—has shown potential in supporting CRC management and general oncologic recovery.

Dietary fiber is a key determinant of gut microbiota composition. Fermentation of fiber in the colon produces Short-Chain Fatty Acids (SCFAs)—such as acetate, propionate, and butyrate—which enhance mucosal integrity, suppress inflammation, and inhibit tumor proliferation (55). High-fiber diets also reduce carcinogenic secondary bile acids and support beneficial microbial populations.

Personalized nutrition, tailored to the unique microbial profile of CRC patients, has been proposed as a preventive and therapeutic strategy (56). Specially formulated diets rich in vegetables, fruits, oilseeds, low-sugar complex carbohydrates, and unsaturated fatty acids offer antioxidant and anti-inflammatory benefits (57, 58). Microecological formulas incorporating short peptides, Lactobacillus, Bifidobacterium, and herbal extracts have demonstrated immune-enhancing and anticancer potential, though further validation is needed to identify microbial or metabolic predictors of dietary response (59, 60).

Probiotics play a restorative role in gut barrier function, immune modulation, and malnutrition correction. Butyrate-producing strains, for example, alleviate intestinal wall atrophy in malnourished tumor patients (61). FMT from healthy donors has shown efficacy in resolving therapy-induced complications, such as Clostridium difficile infections, by restoring microbial diversity (62). Notably, FMT from immune checkpoint inhibitor-responsive donors enhanced PD-1 blockade efficacy in germ-free mice, associated with elevated Akkermansia muciniphila levels (63).

Moderate exercise (e.g., yoga, swimming, walking) has been linked to increased microbial diversity and SCFA production, enhancing gut barrier integrity and immune responsiveness (64–66). Disruption of circadian rhythms negatively impacts microbiota structure and immune balance. Taurocholic acid metabolism, epigenetically regulated by microbial activity, can promote MDSC accumulation and lung metastasis in CRC models when circadian patterns are disturbed (67).

Microbiota-targeted rehabilitation represents a promising adjunct in cancer care, extending beyond tumor suppression to systemic recovery. Future research should prioritize stratified approaches based on microbiome profiling, define optimal combinations of diet, probiotics, and lifestyle interventions, and explore their synergy with frontline oncologic treatments. These strategies not only support tumor rehabilitation but may contribute broadly to patient resilience and survivorship (**Figure 1**).

## 3 Mechanisms by which gut microbes affect tumor therapy

### 3.1 Gut microbes intervene in tumor cell metabolism and metastasis through flora metabolites

A growing body of evidence suggests that gut microbial metabolites play a pivotal role in modulating tumor cell behavior, influencing both therapeutic response and metastatic potential (45, 68–71)(**Table 1**).

**Table 1:** Gut microbes associated with cancer therapies and mechanisms.

Therapy Type	Key Microbes	Mechanisms	Associated Cancers	References
Radiotherapy	*Lactobacillus* spp., *Bifidobacterium* spp.	Alleviate radiation-induced mucositis and diarrhea; enhance epithelial integrity; anti-inflammatory cytokine modulation	Colorectal, Head and Neck	(31, 158)
	*Enterococcus faecalis*, others	Promote IL-1β secretion, tight junction disruption, enhance radiation toxicity via dysbiosis	Colorectal	(159)
Chemotherapy	*Bacteroides fragilis*, *Mycobacterium polymorphum*	Promote Th17/Th1 activation via translocation; enhance cyclophosphamide efficacy	Breast, Colorectal	(160)
Targeted Therapy	*Bifidobacterium* spp., *Prevotella* spp.	Modulate DC activation and T-cell recruitment, enhance trastuzumab response	Breast	(21, 161)
Immunotherapy (ICI)	*Bifidobacterium longum*, *Faecalibacterium prausnitzii*, *Akkermansia muciniphila*	Enhance CD8+ T cell infiltration, improve ICI efficacy via DC activation and IFN-γ production	Melanoma, NSCLC, CRC	(162)
	*Fusobacterium nucleatum*	Suppresses ICI efficacy via TIGIT binding, succinate-mediated IFN-β inhibition	Colorectal	(163)
TCM	*Mycobacterium avium*, *Bifidobacterium* spp.	Modulate bile acid metabolism and interferon-related metabolites; adjust intestinal flora	Liver, Colorectal	(164, 165)
Rehabilitation (Diet, FMT, Probiotics)	SCFA-producing bacteria (*Roseburia*, *Butyricicoccus*, etc.)	Enhance gut barrier, reduce systemic inflammation, promote immune response	Colorectal	(166)

One representative example is indole-3-acetic acid (3-IAA), a tryptophan-derived metabolite produced by Bacteroides fragilis and B. polymorphicus, which was enriched in pancreatic ductal adenocarcinoma (PDAC) patients who responded to chemotherapy. Exogenous 3-IAA supplementation or a high-tryptophan diet enhanced therapeutic efficacy, highlighting its potential as a microbial co-adjuvant in treatment. Similarly, reuterin, secreted by Lactobacillus reuteri, has shown anti-cancer properties in colon cancer models by inducing protein oxidation and suppressing ribosome biogenesis, thereby inhibiting tumor progression (72).

In contrast, some metabolites may promote tumor development. For example, indole-3-acrylic acid (IDA), mainly produced by Streptococcus species enriched in CRC patients, was shown to accelerate CRC progression in mice by inhibiting ferroptosis via the AHR–ALDH1A3 pathway, a mechanism associated with poor prognosis (73).

In addition to soluble metabolites, microbial extracellular vesicles (EVs) can directly modulate tumor behavior. Fn-OMVs, derived from clostridium nucleatum, promote lung metastasis by activating autophagic flux in tumor cells. Inhibiting autophagy with chloroquine significantly reduced metastases in murine models, confirming the pro-metastatic role of microbial vesicles in CRC (74).

These findings underscore a dualistic role of microbial metabolites in cancer: some exert therapeutic potential, while others facilitate tumor progression. The regulatory landscape is shaped by metabolite structure, producing species, and host context. Understanding these mechanisms provides opportunities to: Identify metabolite biomarkers predictive of treatment response; Design diet or microbiota-based interventions to modulate metabolite production; Target pathogenic metabolite pathways, such as ferroptosis suppression or autophagy activation.

**Figure 2:**
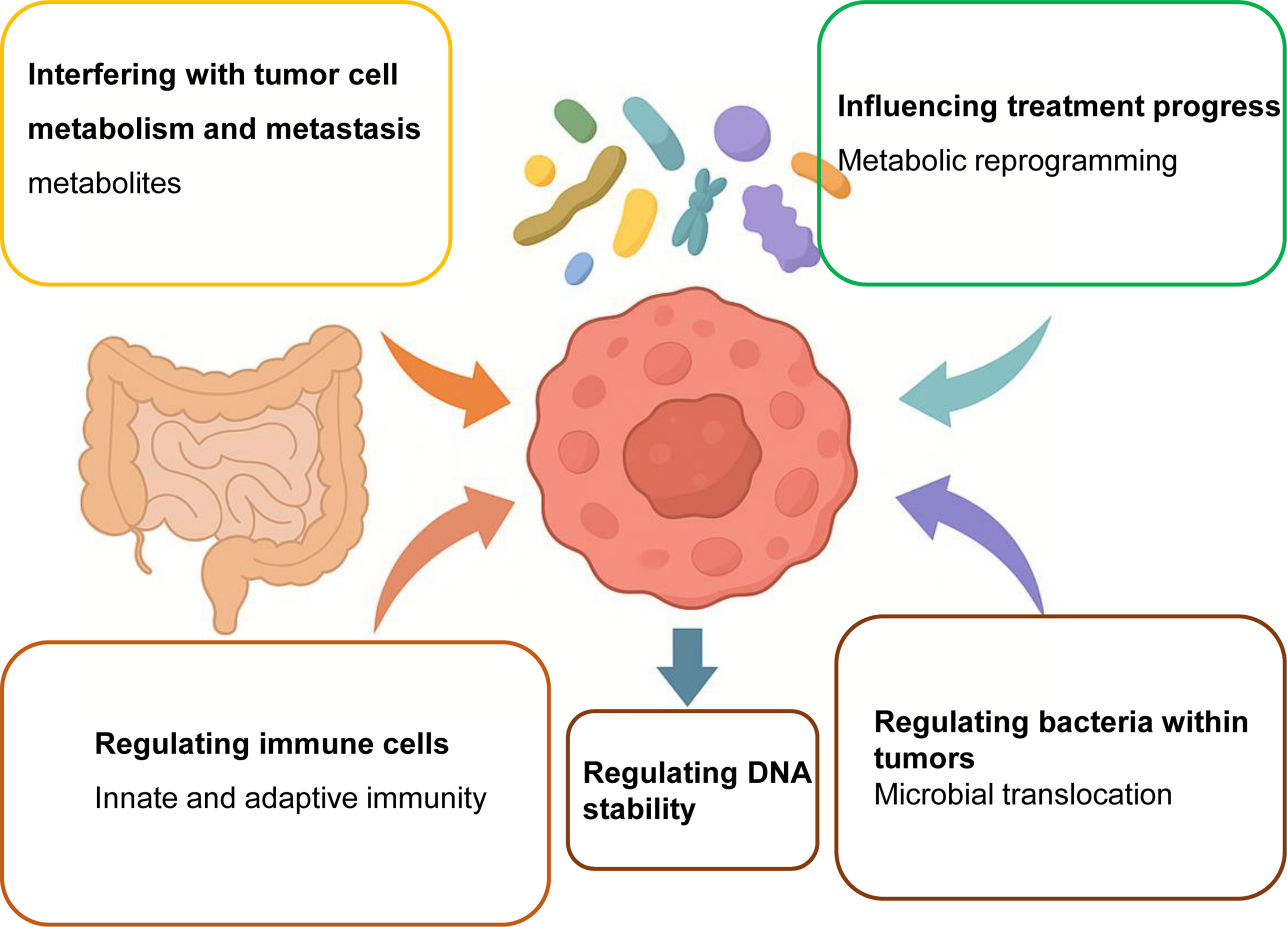
Gut microbes modulate the efficacy of cancer therapies through multiple mechanisms. Gut microbiota exerts a profound influence on cancer therapy outcomes by orchestrating a range of biological processes within the host and tumor microenvironment. These mechanisms include: (1) metabolic reprogramming of tumor cells via microbial metabolites such as SCFAs and tryptophan derivatives, which can either enhance or impair therapeutic efficacy; (2) modulation of the tumor immune microenvironment by influencing T-cell function, antigen presentation, and immune checkpoint expression; (3) remodeling of tumor biomechanical properties such as extracellular matrix stiffness and intercellular adhesion, thereby affecting tumor invasion and immune infiltration; (4) translocation of bacteria from the gut to tumor tissue, contributing to the formation of intratumoral microbiota that can interact with local immune cells; and (5) induction of DNA damage and genomic instability through genotoxic bacterial products like colibactin. Together, these pathways illustrate the multifaceted regulatory roles of gut microbes in shaping therapeutic response, toxicity, and resistance, suggesting new opportunities for microbiota-targeted adjuvant interventions.

Future therapeutic strategies may involve precision modulation of microbiota-derived metabolites, either by microbial engineering or metabolite-mimicking drugs, to enhance antitumor efficacy while mitigating metastatic risk (**Figure 2**).

### 3.2 Gut microbes influence tumor cell therapeutic progression by regulating the metabolic reprogramming of tumor cells

Metabolic reprogramming is a hallmark of cancer, underpinning genomic instability, inflammation, and immune escape (75). As a “second genome,” the gut microbiota play a crucial role in modulating host metabolic pathways, including glucose, lipid, and amino acid metabolism, thereby indirectly reshaping tumor cell behavior and response to therapy (76, 77).

Enhanced glycolysis (the Warburg effect) in CRC leads to lactate accumulation and acidification of the tumor microenvironment (TME), fostering tumor progression (78, 79). Recent studies have linked this metabolic shift to specific gut microbes. Clostridium nucleatum and Clostridium perfringens are enriched in CRC tissues with elevated glucose metabolism, as confirmed via PET/CT imaging and qPCR. Mechanistically, C. perfringens promotes glycolysis by epigenetically modulating histone acetylation through lncRNA ENO1-IT1-mediated regulation of ENO1, a glycolytic enzyme, revealing a microbe-epigenetic-metabolism axis in CRC (80, 81).

Lipid biosynthesis is often upregulated in tumor cells. Circulating tumor-derived lipids can alter intestinal microbiota by damaging bacterial membranes and shifting microbial composition, leading to dysbiosis and inflammation (82). Conversely, gut microbial metabolites such as butyrate and propionate activate PPARγ signaling, which promotes lipid catabolism, reduces hepatic fat accumulation, and may counteract tumor-driven metabolic shifts (83).

Although the role of microbial fatty acids in tumor lipid anabolism remains unclear, they represent promising candidates for therapeutic exploitation (84).Tumor cells exhibit glutamine addiction under glycolytic stress, relying on glutaminolysis for ATP, nucleotide, and redox homeostasis. While direct microbial regulation of glutamine metabolism in tumors is poorly defined, studies suggest that glutamine supplementation can reshape gut microbial communities—reducing the Firmicutes/Bacteroidetes ratio—and elevate protective secretory IgA (SIgA), which maintains intestinal immune integrity (85, 86).Disruption of this axis may promote the leakage of harmful microbial metabolites into circulation, fueling tumor progression.

The gut microbiota influence tumor metabolism through bidirectional nutrient and metabolite exchange. Key microbial products—including SCFAs and lncRNA-regulated epigenetic modifiers—affect tumor energy metabolism, redox balance, and epigenetic landscape. Future research should focus on: Identifying microbe-metabolite-target networks driving therapeutic resistance; Modulating microbial composition to reverse oncogenic metabolic states; Integrating microbiota data into metabolic precision oncology platforms. These insights may unlock novel microbial adjuvants or diet-microbiome interventions that synergize with metabolic-targeted cancer therapies (**Figure 2**).

### 3.3 Gut microbes regulate immune cells in the immune microenvironment

The TME often displays strong immunosuppressive features that hinder the effectiveness of cancer immunotherapies. Emerging evidence indicates that gut microbiota can reshape the immune landscape of the TME by influencing both innate and adaptive immunity, thereby modulating therapeutic response (33, 49, 87–89).

Gut microbes release microbe-associated molecular patterns (MAMPs)—such as lipopolysaccharides, flagellin, and peptidoglycans—that are sensed by host pattern recognition receptors (e.g., TLRs, NOD-like receptors), influencing both systemic and intratumoral immune responses (21, 82, 90). For instance, dietary fiber enhances cyclic di-adenosine secretion from gut microbes, which activates the STING pathway and promotes type I interferon production in the TME, facilitating antigen presentation and boosting the efficacy of immune checkpoint blockade (ICB) therapies (91, 92).

Additionally, microbial sensing impacts therapy-induced immunotoxicity. For example, TLR9 or MYD88 deficiency reduces graft-versus-host disease (GVHD) in mice, while TLR2 signaling attenuates methotrexate toxicity by inducing compensatory metabolic pathways (17, 93).

Increased gut permeability during tumor progression or therapy allows translocation of live bacteria and metabolites into secondary lymphoid tissues or tumors, influencing immune activation (84). Fusobacterium nucleatum (Fn), a CRC-associated microbe, has been shown to localize preferentially in tumors and interfere with ICB efficacy. Fn-derived succinic acid suppresses IFN-I signaling and CD8^+^ T-cell infiltration, while its surface protein FAP2 binds TIGIT on T/NK cells to inhibit antitumor immunity (94–96).

SCFAs such as butyrate, acetate, and propionate, as well as tryptophan metabolites, play key roles in modulating immune responses (97, 98). Butyrate enhances CD8^+^ T-cell activation by inhibiting histone deacetylases and inducing ID2 expression, thereby improving responses to ICB and radiotherapy (99). However, it may also suppress type I IFN production in dendritic cells, reducing radiotherapy efficacy in certain contexts (100).

In contrast, microbial SCFAs also exhibit cytoprotective effects, alleviating treatment-related toxicity. For example, butyrate from Prevotella loescheii mitigates cardiotoxicity associated with PD-1/PD-L1 blockade (101), while propionate and indole-derived metabolites reduce hematologic and gastrointestinal toxicity during radiotherapy (102).

Gut microbiota also influences immune checkpoint expression. Coprobacillus cateniformis, for example, downregulates PD-L2 in dendritic cells, enhancing CD8^+^ T-cell–mediated antitumor immunity. In germ-free mice, blockade of PD-L2 or its receptor RGMb restores responsiveness to anti-PD-1/PD-L1 therapy, in a MYD88-dependent manner (103).

The gut-tumor immune axis is a dynamic interface with both therapeutic potential and challenges. While some bacteria promote immune activation, others contribute to therapy resistance or immune suppression. Conflicting findings across studies highlight the influence of host genetic background, tumor type, and microbiota composition.

Identifying key microbial species and metabolites that predict or modulate immunotherapy outcomes; Targeting microbial pathways (e.g., SCFA production, MAMP sensing) to enhance immune responses; Designing microbiome-informed immunotherapy protocols, including prebiotic/probiotic combinations or microbial-derived adjuvants (**Figure 2**).

### 3.4 Regulation of the biophysical properties of the tumor microenvironment

The biophysical landscape of the TME—including matrix stiffness, cellular adhesion, and mechanical stress—plays a crucial role in cancer progression and immune regulation. Recent studies reveal that gut microbiota, particularly specific bacterial species and their bioactive factors, can significantly influence these mechanical properties, thereby modulating tumor invasiveness and therapeutic response.

In CRC, Fusobacterium nucleatum (Fn) has been shown to enhance tumor cell adhesion to endothelial cells via upregulation of ICAM1, a key adhesion molecule, facilitating extravasation and metastasis in *in vivo* models. This process is mediated by the ALPK1-NF-κB signaling axis (104).Furthermore, circulating tumor cells experience fluid shear stress and other mechanical forces that can cause cytoskeletal damage and limit metastatic potential (105, 106). However, cancer cells invaded by certain bacteria—termed intratumoral microbes (InTM)—exhibit enhanced survival under these conditions. In murine models, bacterial invasion induced RhoA-ROCK-actin remodeling, which strengthens cytoskeletal integrity and increases resistance to mechanical stress, promoting distant metastasis (107).

The exact microbial trigger for this phenotype remains under investigation, but bacterial factors such as C3 ribosyl transferase from Clostridium botulinum, known to alter actin dynamics, are potential candidates (108). Microbial dysbiosis also influences extracellular matrix (ECM) dynamics within tumors. For instance, reduced abundance of Faecalibacterium prausnitzii in CRC patients correlates with enhanced MMP2 activation, leading to chronic inflammation, fibrosis, and excessive ECM deposition (109). These changes result in increased TME stiffness, which has been observed across multiple cancers (e.g., breast, liver, pancreatic, prostate) and is associated with poor prognosis, immune evasion, and therapeutic resistance (105).The stiffened ECM promotes tumor progression through mechanotransduction pathways, enabling cancer cells to sense and respond to physical cues via integrins and YAP/TAZ transcriptional regulation, further reinforcing invasive phenotypes.

Gut microbes not only influence biochemical signaling but also remodel the physical architecture of the TME through: Modulation of adhesion molecules (e.g., ICAM1); Cytoskeletal reprogramming under shear stress; Fibrosis-induced ECM stiffening. These biomechanical changes contribute to metastasis, immune suppression, and therapy resistance. Understanding how specific microbial species interact with tumor biophysics offers a novel therapeutic avenue. Targeting bacteria-ECM-cytoskeleton crosstalk may allow us to “soften” the TME and enhance antitumor immunity or drug penetration (**Figure 2**).

### 3.5 Gut microbes influence intracellular bacteria in tumors to regulate tumor development

Studies have increasingly demonstrated that microorganisms can negatively impact antitumor immunity, particularly in pancreatic cancer. In a genetically engineered mouse model of PDAC, Pushalkar et al. identified a high abundance of bacteria within tumor tissues, with up to 20% originating from the gut microbiota—an observation further supported by human surgical specimens in which these bacteria were absent from adjacent non-tumor areas (110, 111). Notably, pancreatic tumorigenesis was found to induce time-dependent disruptions in gut microbial composition, which correlated with Kras activation, highlighting a dynamic gut-tumor microbiome interaction during disease progression (112, 113).

Beyond direct colonization, the intratumoral microbiome can also be shaped indirectly by gut microbiota modulation. FMT from short-term survivors (STS), long-term survivors (LTS), and healthy individuals into tumor-bearing mice led to distinct differences in tumor microbiome composition, immune infiltration, and tumor growth, underscoring the profound influence of gut-derived microbial signals on the tumor immune microenvironment (17, 114).

The origins of the intratumoral microbiota are believed to include mucosal surfaces (e.g., gastrointestinal tract, lungs), nearby normal tissues, and the circulatory system (115). Tumors at mucosal sites are particularly susceptible to microbial infiltration due to barrier dysfunction. For example, PDAC-associated bacteria have been shown to translocate from the gut to the pancreas via the pancreatic duct, facilitated by the unique inflammatory and immunosuppressive microenvironment of adenocarcinoma (116, 117). However, even tumors in non-mucosal sites such as the breast harbor microorganisms, suggesting alternative routes of microbial entry, possibly via blood or immune cells (118).

A 2020 study further revealed that tumor-resident microbiota closely resembles the bacterial communities in adjacent normal tissues, suggesting NATs as a potential seeding source for intratumoral bacteria (119). Given the multisource and tissue-specific nature of tumor microbiota, systematic comparisons across tumor types and anatomical regions may help uncover tumor-specific microbial signatures. These findings open new avenues for cancer prevention and precision therapeutics by targeting microbial components of the tumor microenvironment (**Figure 2**).

### 3.6 Regulating DNA stability

Gut microbes have emerged as key players in CRC pathogenesis through their capacity to induce genotoxic stress, compromise genome integrity, and drive malignant transformation (120–122). One of the most well-characterized mechanisms involves colibactin, a genotoxin synthesized by pks+ strains of Escherichia coli, which causes DNA double-strand breaks, triggers DNA damage response cascades, and increases chromosomal instability and mutation frequency. *In vivo* experiments have shown that inhibiting colibactin production can suppress tumor development, underscoring its critical role in carcinogenesis.

Furthermore, colibactin-producing E. coli can act synergistically with enterotoxin-producing Bacteroides fragilis-like species. These bacteria degrade the protective mucus barrier, facilitating the colonization of the colonic mucosa by pks+ E. coli, thereby amplifying DNA damage within epithelial cells. This coordinated microbial assault accelerates neoplastic initiation in the colon.

To explain these dynamics, researchers have proposed the “driver–passenger” model: early “driver” bacteria possess oncogenic traits that initiate DNA damage and tumor formation. As tumor progression remodels the local microenvironment—through inflammation, nutrient shifts, and immune suppression—it becomes more permissive to “passenger” or opportunistic bacteria. These later colonizers, although not directly oncogenic, benefit from the altered niche and further exacerbate tumor development through immune modulation or metabolic interactions (31, 123).

In summary, gut microbes contribute to CRC not only by initiating genotoxic events but also by participating in a dynamic ecological succession that sustains and promotes tumor progression. This interplay highlights the potential of targeting specific microbial signatures or their genotoxins as a strategy for CRC prevention and intervention (**Figure 2**).

Recent studies have further elucidated the diverse mechanisms by which gut microbes influence cancer progression and therapeutic responses. Notably, the STING signaling pathway has emerged as a critical mediator linking microbial-derived signals to innate immune activation, thereby shaping antitumor immunity (124). Additionally, autophagy regulation has been shown to intersect with microbial cues, affecting tumor cell survival and responsiveness to treatment (125). Microbial metabolites also play pivotal roles in driving epigenetic reprogramming within the tumor microenvironment, altering gene expression patterns that can either suppress or promote tumorigenesis (15). Moreover, Bifidobacterium species have been reported to enhance dendritic cell maturation and T-cell activation, strengthening host antitumor immune responses (126). Together, these insights underscore the multifaceted ways in which gut microbes and their metabolites modulate cancer biology and highlight promising avenues for therapeutic intervention.

## 4 Microbiota-targeted therapeutics: clinical perspectives and challenges

### 4.1 Current clinical applications of FMT, probiotics, and next-generation probiotics

In recent years, microbiota-targeted interventions have been increasingly explored in cancer therapy, with FMT emerging as a promising strategy for microbiota reconstruction. FMT has shown preliminary clinical value in modulating immunity and enhancing therapeutic efficacy (127–131). Particularly in patients with poor responses to ICIs, FMT has been demonstrated to restore microbial diversity and metabolic function, thereby improving the immune microenvironment and therapeutic outcomes (**Table 2**). For instance, FMT from ICI-responsive donors has been applied to patients with melanoma and non-small cell lung cancer, leading to significant alterations in gut microbial composition and immune cell infiltration (132). Additionally, probiotics and next-generation probiotics have exhibited potential in supporting radiotherapy, chemotherapy, or ICI therapy in several phase I/II clinical trials (133, 134). However, current applications are largely empirical, lacking standardized and personalized clinical protocols. The substantial functional heterogeneity among strains, challenges in maintaining formulation viability, and the complex clinical backgrounds of recipients all obscure the translational pathway. Therefore, future efforts should focus on identifying functional microbial biomarkers and elucidating underlying mechanisms to advance gut microbial interventions from empirical approaches toward mechanism-driven precision strategies.

**Table 2:** Summary of gut microbiota-related studies in cancer therapy.

Therapy Type	Microbial Species	Model System	References
Immunotherapy	*Bifidobacterium* spp.*, Akkermansia muciniphila*	Clinical, Animal	(146, 167, 168)
Immunotherapy	*Fusobacterium nucleatum*	*In vitro*, Animal	(169)
Chemotherapy	*Fusobacterium nucleatum*	Animal	(81)
Radiotherapy	*Lactobacillus rhamnosus GG*	Animal	(31)
Traditional Chinese Medicine	*Bifidobacterium* spp.	Animal	(170)
FMT	*Donor microbiota*	Clinical	(36)

### 4.2 The potential of synthetic biology-engineered bacteria and phage therapy in cancer treatment

With advances in synthetic biology and microbial engineering, engineered bacteria and phage therapy have opened unprecedented avenues in cancer treatment (135, 136). By genetically modifying gut-colonizing bacteria such as Escherichia coli, it is now possible to endow them with the ability to selectively release cytokines, immune-activating molecules, or anti-tumor metabolites within the tumor microenvironment—effectively functioning as an “*in vivo* micro-factory” for targeted therapy (137, 138). For instance, engineered *E. coli* Nissle strains have been developed to express PD-L1 nanobodies, with the potential to enhance the penetration and efficacy of immune checkpoint therapies (137). In addition, synthetic phages can be designed to selectively eliminate oncogenic bacterial populations (e.g., Fusobacterium nucleatum), thereby mitigating their roles in promoting cancer cell adhesion and immune suppression (139, 140).

However, several technical challenges remain in the clinical translation of these strategies, including biosafety concerns, microbial co-adaptation, and the risk of genetic drift. Achieving precise control over *in vivo* activity—such as spatiotemporal release, modulation of immune tolerance, and avoiding unintended disruption of the gut microbiota—remains a critical bottleneck. Moving forward, integrating dynamic simulation modeling, nanocarrier delivery platforms, and CRISPR-based regulatory systems is expected to enhance the precision, controllability, and clinical viability of these synthetic microbiome-based interventions.

### 4.3 Controllability, safety, and interindividual variability in microbiota-based interventions

Although microbiota-targeted interventions have demonstrated promising therapeutic effects, significant interindividual variability remains one of the primary barriers to clinical translation (33, 127). The gut microbiota exhibits highly personalized characteristics influenced by factors such as genetic background, dietary patterns, antibiotic usage history, and baseline immune status, leading to substantial differences in response to the same microbial intervention across individuals (141–143). Furthermore, the diffusion, retention time, and interactions of FMT and engineered microbial preparations during intervention are complex and can lead to unpredictable efficacy, immune responses, or dysbiosis (144).

In terms of safety, there is currently a lack of systematic assessment regarding the potential pathogenicity and cumulative toxicity associated with the long-term use of engineered bacteria or FMT (143, 145, 146). Serious infection events reported in some clinical cases of FMT have also exposed shortcomings in donor screening and risk management protocols. Therefore, establishing standardized recipient/donor matching criteria, predictive models for pre-intervention microbiota structure and function, and incorporating dynamic monitoring alongside pharmacokinetic profiling are crucial strategies to enhance the reliability and safety of microbiota-based cancer interventions.

### 4.4 Challenges in standardized modeling and multi-omics integration

The lack of standardization and integration of multi-omics data significantly hampers the clinical advancement of microbiota-based therapies. In clinical settings, microbiota sequencing and functional prediction often suffer from inconsistencies in data dimensions, non-uniform analytical methods, and poor reproducibility, making cross-study comparisons and longitudinal accumulation of evidence difficult (147). Moreover, current intervention strategies lack mechanisms for synergistic interpretation with other omics data (e.g., metabolomics, transcriptomics, proteomics), which hinders the establishment of clear causal links between microbiota changes and therapeutic outcomes (148).

Although some studies have identified specific microbial taxa associated with treatment responses, elucidating their signaling pathways or the role of microbial metabolites remains a challenge (20, 133, 149–151). Therefore, future efforts should focus on developing standardized microbiota intervention models, unifying protocols for sampling, sequencing, and analysis, and integrating clinical cohort data with mechanistic studies. Leveraging big data platforms to build a cross-omics analytical framework will be crucial to strengthen the evidence base and provide traceable mechanistic insights for microbiota-targeted therapies.

### 4.5 AI and microbiome integration for therapeutic prediction and precision intervention design

Encouragingly, the integration of artificial intelligence (AI) technologies offers a novel approach to achieving precision microbiome interventions. AI models can extract features and uncover associations within the complex tripartite relationship between the microbiota, host, and therapy, enabling predictive modeling of key microbial taxa linked to therapeutic responses (152, 153). On this basis, the incorporation of multi-omics datasets—such as scRNA-seq, metabolomics, and 16S rRNA sequencing—allows AI to optimize personalized intervention strategies and enhance both therapeutic efficacy and safety margins (154, 155).

Recent studies have demonstrated that deep learning can identify characteristic microbial signatures in responders to ICIs, thereby supporting donor selection and improving the predictive accuracy of FMT outcomes (156). Furthermore, reinforcement learning algorithms can simulate the impact of different intervention pathways on microbial succession, assisting in the selection of intervention targets and the timing of therapeutic decisions (157).

However, current AI systems remain limited by the scale of training data, the precision of microbial taxonomic annotation, and insufficient causal inference capabilities. Moving forward, it is essential to leverage multicenter clinical cohorts and construct high-quality, well-annotated datasets. The development of interpretable and generalizable AI models will be critical to achieving an intelligent leap from population-level microbial “common pattern recognition” to truly individualized microbiome regulation.

## 5 Conclusion and perspectives

In recent years, gut microbiota has emerged as a crucial player in host immune modulation and metabolic regulation, gaining increasing prominence in the field of cancer therapy. A growing body of evidence indicates that the composition of the gut microbiota, its metabolic products, and its interactions with host cells significantly influence the outcomes of various antitumor treatments, including immunotherapy, chemotherapy, and radiotherapy. From enhancing treatment response rates to alleviating adverse effects and reshaping the tumor microenvironment, gut microbes exhibit a “triple role” of response prediction, therapeutic potentiation, and toxicity mitigation. As such, targeting the gut microbiome has become a new research frontier with the potential to transform next-generation cancer intervention strategies.

Despite impressive progress, the complex mechanisms through which gut microbes affect cancer therapy remain incompletely understood. High interindividual variability—driven by host genetics, immune status, and metabolic profiles—poses a challenge in decoding causative microbial-host interactions from vast multi-omics datasets. Moreover, the phenomenon of microbial “co-morbidity-coexistence-co-therapy” complicates efforts in precise clinical targeting. Current studies largely remain at the level of association analysis, with limited functional validation or mechanistic elucidation, which restricts the clinical application of gut microbiota as reliable biomarkers or therapeutic targets.

The controllability and safety of microbial interventions also represent major bottlenecks in translational applications. Existing strategies such as FMT, probiotic/next-generation microbial formulations, and synthetic engineered bacteria have shown therapeutic promise to some extent. However, issues such as poor stability, undefined side effect profiles, and potential interference with host immunity and metabolism persist. This is especially critical in the context of cancer, where patients often exhibit compromised immune systems, narrowing the “therapeutic safety window.” Therefore, intervention strategies must be accompanied by enhanced precision and controllability under dynamic immunological conditions.

**Figure 3:**
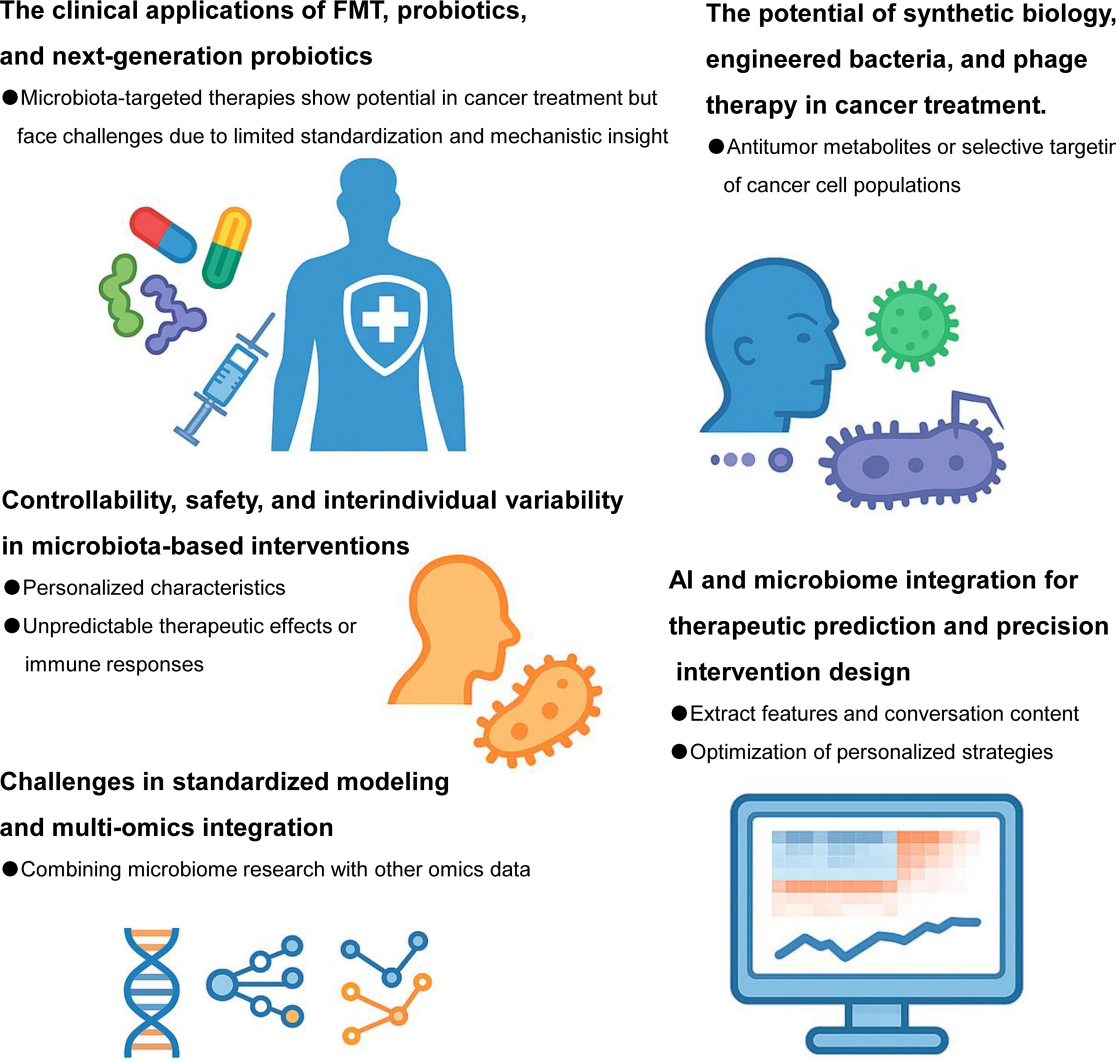
Future directions for gut microbes in cancer therapy. Harnessing the potential of gut microbes represents a promising frontier in precision oncology. Future research should focus on identifying key microbial taxa that influence specific anti-tumor therapeutic modalities—including chemotherapy, radiotherapy, immunotherapy, and targeted therapies—through detailed microbiome profiling. Mechanistic studies are needed to elucidate how gut microbes and their metabolites modulate treatment response, therapeutic resistance, and host immunity. Moreover, robust clinical trials must be conducted to validate the efficacy and safety of microbiota-based interventions such as probiotics, FMT, and microbial metabolite supplementation in cancer patients. With the advancement of synthetic biology and nanotechnology, the design of “artificial anti-tumor bacteria” engineered to deliver drugs, modulate the tumor microenvironment, or boost host immune responses could enable precise and controllable microbial therapy. Ultimately, integrating microbiome science with multi-omics and artificial intelligence tools will drive the development of individualized, microbiota-informed strategies for improving cancer treatment outcomes.

Future research should emphasize the development of an integrated “tumor × microbiome × immunity × metabolism” framework, leveraging single-cell sequencing, spatial multi-omics, and metabolomics to enable in-depth analysis from population-wide to single-cell resolution (**Figure 3**). The incorporation of AI will be pivotal in overcoming existing limitations. AI-driven tools for microbiome prediction modeling, immune response forecasting, and individualized intervention optimization will significantly improve the clinical interpretability of microbiome data, fostering the evolution from “experience-based” to “mechanism-driven precision microbiome therapy.”

Ultimately, personalized microbiome-based therapy is poised to become a central component of future anticancer strategies. Given the regional, dietary, lifestyle, and genetic diversity of microbiomes, constructing high-resolution population microbiota maps and dynamic evaluation systems will be a key research priority. By establishing a three-dimensional interactive network of “microbiota-host-tumor,” it may become feasible to design early screening strategies, personalized interventions, and synergistic treatment pathways based on microbiota status—achieving truly symbiotic and microbiota-empowered anticancer approaches.
